# A New Submersion Detection Sensor Using Two Resistance Temperature Detectors Operating on the Thermal Equilibrium Principle

**DOI:** 10.3390/s19194310

**Published:** 2019-10-04

**Authors:** Youngjun Lee, Young Sam Lee

**Affiliations:** Department of Electrical Engineering, 100 Inha-ro, Michuhol-gu, Inha University, Incheon 22212, Korea

**Keywords:** submersion detection sensor, submersion detector, submersion sensor, RTD, signal conditioning circuit

## Abstract

In this study, a new submersion detection sensor with improved reliability and stability is proposed. The new sensor uses two Resistance Temperature Detectors (RTDs) and operates on the thermal equilibrium principle. The submersion detection sensor controls two RTDs that maintain a constant temperature difference between them in the surrounding environment. The first RTD is used as a reference sensor to measure ambient temperature and the second RTD is supplied with higher current than the reference sensor for self-heating. When submerged, because the thermal conductivity and convective heat transfer coefficient of water are higher than that of air, the temperature difference between the two RTDs is lower in water than in air based on the thermal equilibrium principle. Under these conditions, a submersion detector with a signal conditioning circuit detects these temperature differences. The static performance of the proposed sensor was evaluated by checking whether malfunctions occurred at varying ambient temperatures, differing humidities, and when there was rainfall. In addition, the dynamic performance was evaluated using the response time at varying ambient air temperatures before submersion and with changing water temperatures after submersion, as a metric. The proposed submersion detection sensor is expected to find useful application in aircrafts, ships, and various other industrial fields.

## 1. Introduction

In habitual flood areas, water level measuring systems are required to collect water level information for preventive measures [1]. Furthermore, in the case of fleets of ships, a system that prepares for problems caused by water or oil leakage and fuel shortage by determining the extent of the submersion of the ships, or by measuring the level of fuel in a fuel tank is required [2,3,4]. Therefore, submersion detection sensors are applied in various fields, and a lot of research has been carried out to enhance their reliability.

The submersion detection sensors studied and applied to date mainly utilize the detection of differences in capacitance, electrical resistance, and the refraction or reflection of optical fibers [5,6,7,8,9,10]. However, because submersion sensors detect differences in capacitance on the contact surfaces of the sensors, there may be malfunctions under conditions of high humidity and rainfall. Although the submersion sensor using two electrodes detects the reduced electrical resistance caused by an electrical short circuit when the electrode is submerged, it is difficult to guarantee the sensor’s electrical characteristics and reliability due to the problem of insulation [11,12]. Recently studied fiber optic sensors detect submersion using the difference in the refractive index of air or liquid at the surface of the sensor. Under high humidity or rainfall, however, there may be water on the fiber’s end and foreign objects attached to the end of the fiber that may cause malfunctions [13,14,15,16,17,18,19,20].

In this study, a submersion detection sensor that included a submersion sensor operating on the thermal equilibrium principle and a signal conditioning circuit were proposed in order to solve problems caused by conventional submersion sensors (malfunctions caused by high humidity and rainfall conditions, insulation issues caused by electrical short circuits, and malfunctions due to foreign objects on the sensor). The proposed submersion detection sensor uses two Resistance Temperature Detectors (RTDs) to sense temperature. The first RTD is used as a reference sensor to measure ambient temperature, and the second RTD is supplied with a higher current than the reference sensor for self-heating. The higher current also serves to create and maintain a temperature difference between the RTDs. Under submerged conditions, the thermal conductivity of water is higher than that of air, and the heat transfer coefficient of a fluid is significantly greater than that of air. Thus, if the temperature difference between the RTDs is reduced underwater, submersion is detected in the signal conditioning circuit. In this study, the heat transfer characteristics of a submersion sensor for some range of air temperature and submersion temperature were analyzed with MATLAB, and the static and dynamic characteristics of the sensor were evaluated through experiments [21].

## 2. Materials and Methods

### 2.1. Materials and Sensor Mechanical Design

Two RTDs were used in the proposed submersion detection sensor. The first RTD measured the air temperature and the second RTD was used as a heating sensor. The heating sensor maintains a temperature that is higher than the ambient air temperature by absorbing a higher current. The well-known self-heating characteristic of RTDs is utilized to heat the sensor.

The RTD used in this study was 1PT100GO1020, which was provided by Omega, and is made of glass wire wound platinum, Pt-100, Class B (IEC751), and α = 0.00385 (TCR: Temperature coefficient of resistance). The dependence of the resistance of the RTDs on changing temperature can be expressed in a linear approximation as follows:

(1) $$R_{RTD}=R_{0}\left[1+\alpha \left(T_{i}\right)\right],$$

where $R_{0}$ is the resistance of RTD at 0 °C, 100 Ω, and $\alpha \left(T_{i}\right)$ is the TCR at the measured temperature $T_{i}$. Table 1 shows the response time and self-heating characteristics of the RTD sensor [22].

**Figure 1:**
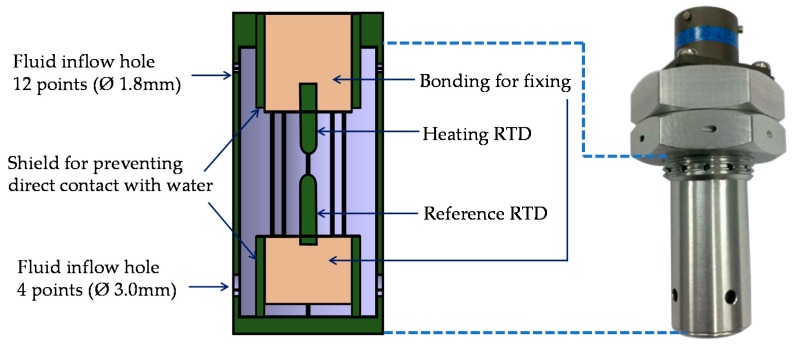
Mechanical design of the proposed submersion sensor.

The submersion detection sensor shown in Figure 1 was designed with the heating RTD and the reference RTD facing one another and used a silicon bond to fix the RTDs. The fluid inflow holes consisted of four ∅ 3.0 mm holes at 90° intervals on the underside and twelve ∅ 1.8 mm holes at 30° intervals on the upper side. The reason behind why the fluid inflow holes were installed on the underside and the upper side was to prevent internal air pockets when submersion occurred vertically and to detect the submersion in all directions. Furthermore, the shield for preventing direct contact with water was structurally designed to prevent malfunctions caused by rainwater. The outer casing was made of aluminum and the total dimensions of the submersion detection sensor were ∅ 20.0 × 50.0 mm where the thickness of the sensor was 50.0 mm.

### 2.2. Transient Heat Transfer Analysis and Signal Conditioning Circuit Design

The operating principle of the submersion detection sensor proposed in this study can be explained theoretically through the analysis of transient heat transfer and a simulation performed with MATLAB.

In an electronic circuit, the temperature of an RTD was measured by the voltage across it. Normally, the RTD is supplied with a low current, several milliampere(mA), in order to minimize the errors that arise from self-heating due to the current flow. However, the submersion detection sensor presented in this study supplied tens of mA to the heated RTD for heating. Furthermore, the reference RTD was supplied with several mA to minimize self-heating. According to the first law of thermodynamics, the heat of the heating RTD can be expressed as follows:

(2) $$\dot{Q}={\dot{Q}}_{i}+{\dot{Q}}_{cv},$$

where $\dot{Q}$ is the heat transfer rate of the heating RTD, ${\dot{Q}}_{i}$ is the heat transfer rate of the heating element heated by the current applied to the resistance element (platinum) inside the RTD, and ${\dot{Q}}_{cv}$ is the convective heat transfer rate that governs the amount of heat escaping into the fluid around the glass. In the case of a solid, a lumped system analysis can be applied to analyze the heat transfer in transient conditions if the Biot number is less than 0.1. The equation to obtain the Biot number is as follows [23]:

(3) $$B_{i}=\frac{hL_{C}}{k},$$

where $B_{i}$ is the Biot number, h is the heat transfer coefficient of fluid [W/m^2^ °C], k is thermal conductivity [W/(m °C)], and $L_{C}$ is the characteristic length of the heat transfer [m]. The value of $L_{C}$ was calculated using the following equation and was 0.0004545 m, assuming that the RTD and surrounding glass body was a single thermal mass.

(4) $$L_{c}=\frac{V}{A}=\frac{Volumetric\;of\;heating\;element}{surface\;of\;heating\;element}=\frac{\pi lr^{2}}{2\pi rl+2\pi r^{2}}.$$

The thermal conductivity k in Table 2 was obtained through experiments carried out in normal and submerged conditions. The value of k in the submerged condition was assumed to be 1.8 times larger than that under normal conditions because the heat was also transferred from the surface of the RTD to water. The calculated values of $B_{i}$ are as follows.

As shown in Table 2, the calculated values of $B_{i}$ were less than 0.1 under all conditions and could be analyzed by the lumped system.

Because the rate of the change in temperature calculated by the lumped system in the heating RTD was equal to the rate of the change in internal energy, it could be expressed as follows:

(5) $$\dot{Q}\left(t\right)=\varrho cV\frac{dT}{dt}=mc\frac{dT}{dt},$$

where ϱ is density, c is specific heat, V is volume, and m is mass. Using Equation (2), $\dot{Q}$ can be rewritten as follows:

(6) $$\dot{Q}={\dot{Q}}_{i}+{\dot{Q}}_{cv}=\frac{I^{2}R}{kL_{C}}+hA\left(T_{i}-T_{\infty }\right),$$

where I is current, and R is the resistance of the RTD. The heat loss of the RTD can be expressed by Newton’s law of cooling. In Equation (6), h is the convective heat transfer coefficient of fluids, A is the heat transfer area of the RTD, $T_{i}$ is the initial temperature (i.e., environmental temperature) of the RTD, and $T_{\infty }$ is the temperature of the fluid at a sufficient distance from the RTD [24,25].

From Equations (5) and (6), we obtained the following differential equation:

(7) $$mc\frac{dT}{dt}=\frac{I^{2}R}{kL_{C}}+hΑ\left(T_{i}-T_{\infty }\right)$$

The solution to Equation (7) could be written as follows:

(8) $$T\left(t\right)=\frac{I^{2}R}{kL_{C}}\left(1-e^{-\frac{kL}{mc}t}\right)+\left(T_{i}-T_{\infty }\right)e^{-\frac{hΑ}{mc}t}+T_{\infty }.$$

The value of the product of the mass, *m* [kg], and the specific heat, *c* [W/(kg°C)], was the inverse of 0.26 [°C /1 mW]. This value was the self-heating error value of the RTD specification presented in Table 1, and was equal to 0.00384615 [W/°C].

**Figure 2:**
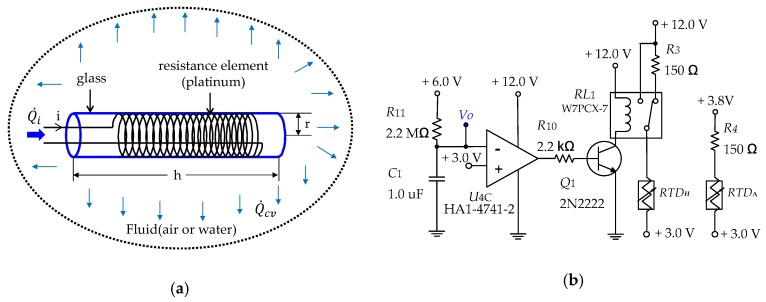
(**a**) Heat transfer diagram of a heating resistance temperature detector; (**b**) signal conditioning circuit.

Figure 2 shows the diagram of the heat transfer occurring in the fluid of the heating RTD and the signal conditioning circuit.

In the signal conditioning circuit, the warm-up circuit consists of $U_{4C}$ (Op amp HA1-4742-2), $R_{11}$, and $C_{1}$. Together, they prevent malfunctions due to high humidity and moist conditions when the system is operating. $V_{o}$, the voltage applied to $U_{4C}$, was as follows:

(9) $$V_{o}\left(t\right)=6\left(1-e^{-\frac{1}{R_{11}C_{1}}t}\right),$$

From above, the time taken for the voltage to reach $V_{o}$ could be written as follows:

(10) $$t=-R_{11}C_{1}\times \ln\left(1-\frac{V_{o}}{6}\right).$$

The calculated t was about 1.52 s with $V_{o}$ set to 3.0 V, $R_{11}$ was 2.2 MΩ, and $C_{1}$ was 1.0 µF. In the case of t < 1.52, the relay $RL_{1}$ was activated by turning Q1 ON, which implies that $RTD_{H}$ was directly connected to a voltage of 12.0 V. In the case of t≥ 1.52, the relay $RL_{1}$ was deactivated by turning Q1 OFF. In this case, $RTD_{H}$ was connected to 12.0 V through $R_{3}$.

Equations (11) and (12) can be derived from the dependence of the resistance of the $RTD_{H}$ on temperature, taking into account the signal conditioning circuit in Equations (1) and (8).

In the case of t < 1.52, the temperature of $RTD_{H}$ was given as follows:

(11) $$T\left(t\right)=\left(\frac{V^{2}}{RTD_{H}\times kL_{C}}\right)\times \left(1-e^{-\frac{kL}{mc}t}\right)+\left(T_{i}-T_{\infty }\right)e^{-\frac{hΑ}{mc}t}+T_{\infty }.$$

In the case of t≥ 1.52, we had

(12) $$T\left(t\right)=\frac{{\left(\frac{V}{R_{3}+RTD_{H}}\right)}^{2}RTD_{H}}{kL_{C}}\times \left(1-e^{-\frac{kL}{mc}t}\right)+\left(T_{i}-T_{\infty }\right)e^{-\frac{hΑ}{mc}t}+T_{\infty }.$$

The temperature of $RTD_{A}$ could be expressed as follows:

(13) $$T\left(t\right)=\frac{{\left(\frac{V}{R_{4}+RTD_{A}}\right)}^{2}RTD_{A}}{kL_{C}}\times \left(1-e^{-\frac{kL}{mc}t}\right)+\left(T_{i}-T_{\infty }\right)e^{-\frac{hΑ}{mc}t}+T_{\infty }.$$

**Figure 3:**
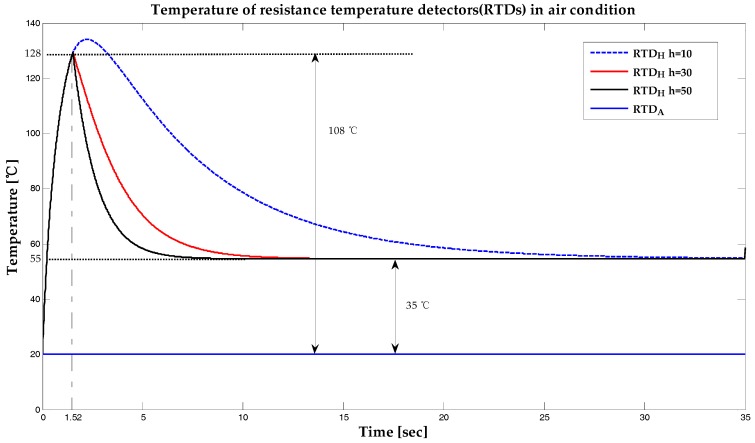
Temperature of resistance temperature detectors (RTDs) when surrounded by air.

Based on Equations (11), (12), and (13), the results of the simulation performed using MATLAB for the temperature of each RTD when it was surrounded by air is presented in Figure 3.

**Figure 4:**
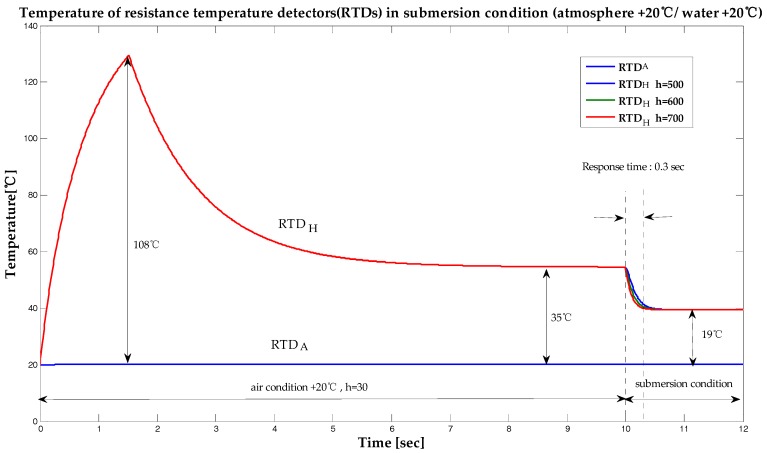
Temperature of resistance temperature detectors (RTDs) under submerged conditions (atmosphere + 20 °C / water + 20 °C).

In Figure 3, the purpose of heating the $RTD_{H}$ within the initial 1.52 s was to increase the ambient temperature by about 108 °C in order to prevent possible malfunctions when the RTD was powered ON in wet conditions, and to reduce warm-up time. After 1.52 s, the current supplied to the $RTD_{H}$ was reduced. This, in turn reduced the temperature difference between the $RTD_{H}$ and the surrounding air to about 35 °C. Next, the $RTD_{A}$ was simulated to be almost the same as the air temperature. The values of the convective heat transfer coefficient of air, *h*, ranging from 10 to 50 were simulated, and it took 25 and 5 s to reach thermal equilibrium for heat transfer coefficient values of 10 and 50, respectively. Figure 4 shows the changes in temperature between these RTDs under submerged conditions. This simulation result makes it easy to understand the principle behind the operation of the proposed sensor.

**Figure 5:**
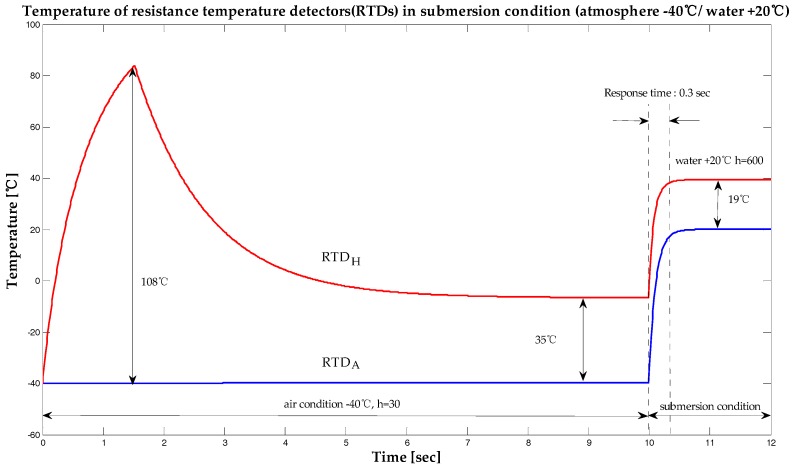
Temperature of resistance temperature detectors(RTDs) under submerged conditions (atmosphere −40 °C/ water +20 °C).

**Figure 6:**
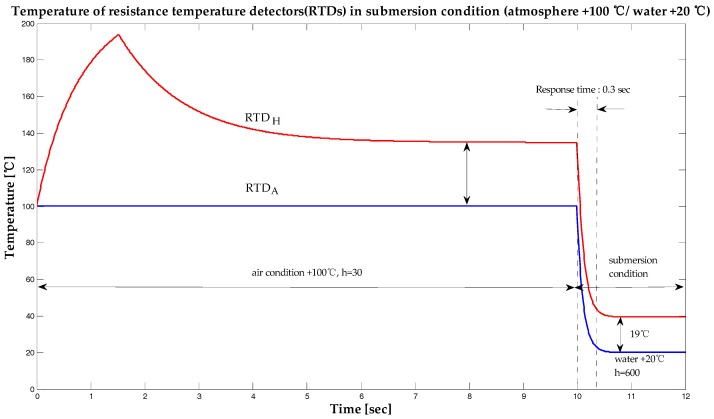
Temperature of resistance temperature detectors (RTDs) under submerged conditions (atmosphere +100 °C / water +20 °C).

In order to understand easily the changing characteristics of the RTD with varying temperatures, the submersion temperature and the air temperature were both simulated under the same temperature of 20 °C. Submersion was carried out 10 s after the experiment commenced. The temperature difference between the RTDs decreased from 35 °C to about 19 °C (55%) 0.3 s after submersion. Although water has a convective heat transfer coefficient of about 600, three different heat transfer coefficient values of 500, 600, and 700 were simulated to allow for a sufficiently large margin of error. The response time of the submersion sensor was simulated to be 0.3 s. The results of the simulations of the submersion sensor performed with a submersion temperature of 20 °C and under atmospheric ambient temperatures varying from −40 °C and 100 °C are presented in Figure 5 and Figure 6 respectively. As shown in these figures, the temperature of $RTD_{H}$ was not affected by changes in the temperature of the environment.

**Figure 7:**
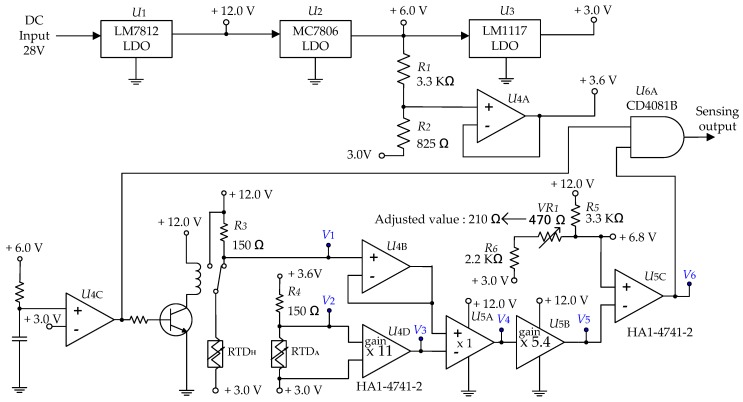
Simplified circuit to detect submersion.

Based on the simulation results, the simplified circuit for implementing the submersion detection is shown in Figure 7. In order to detect very small voltages from the temperature difference between the RTDs, the power supply circuit was designed using components LM7812 (±4 % at T=25 ℃), LM1117(± 1 % at T = 25 ℃) from Texas Instrument Inc and MC7806(±1.5 % at T = 25 ℃) from ON Semiconductor that were low drop voltage linear regulators with low noise. In addition, the component HA1-4741-2 manufactured by INTERSIL was used to precisely detect low voltages. HA1-4741-2 is an Op-amp that has a low input offset feature (3 mV at T=25 ℃). The reason for using VR1(Trimmer) was to solve the problem caused by device tolerance.

The simulation and measurement results of the temperature, resistance, and Op-amp output voltage of the RTDs under the same air and submersion temperature of 20 °C are presented in Table 3.

The test results in Table 3 show that the actual measurements were similar to the simulated values. From Table 3, the measured value of $V_{1}$ changed from 7.04 V to 6.92 V when submerged. This difference value of 0.11 V was too small to be used as a detection signal. Therefore, the signal monitoring circuit that uses an Op-amp was designed so that the voltage of $V_{5}$ can be amplified to 0.67 V for easy recognition. The comparator of $U_{5C}$ was used to generate the final output and the reference voltage of the $U_{5C}$ was configured to be 6.78 V. Under normal operating conditions (when surrounded by air), the output voltage is 7.04 V, and the sensing output is 0 V (“low”). In the submerged condition, the output voltage was 6.92 V, and the sensing output was 12 V (“high”). The error margins of the sensing voltages depend on the external conditions. They were configured to be 0.45 V under the normal condition of being surrounded by air and 0.21 V in the submerged condition, respectively. They were designed to have sufficient margins to prevent malfunctions caused by noise and the tolerance of the component. After the power was turned on, because the voltage difference between the RTDs raised slowly during the initial heating procedure, the sensing output circuit used the CD4081B AND gate from Texas Instruments to prevent submersion detection during the initial 1.52 s period. The reason behind why the design was based on logic gates instead of a μ-controller was to exclude the effect of EMI (Electromagnetic Interference).

### 2.3. Self-Diagnosis Circuit

**Figure 8:**
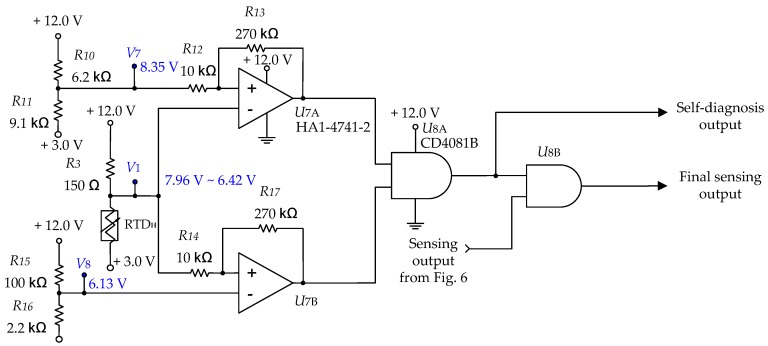
Part of the self-diagnosis circuit for detecting malfunctions.

The submersion sensor and detector were composed of RTDs and electronic circuits, and a self-diagnosis circuit was configured to detect malfunctions caused by bad RTDs or problems with electronic circuits. The circuit illustrated in Figure 8 shows a portion of the self-diagnosis circuit. It checks the voltages in each major circuit to verify that the voltage at each terminal is in the normal range by processing the signals using the AND gate.

The ambient temperature of the operational environment of the submersion detector is required to be in the range −40 °C to +100 °C. Because the $RTD_{H}$ is always 35 °C higher than the $RTD_{A}$, the minimum temperature of the $RTD_{H}$ should be −5 °C but was assigned a value of −20 °C to allow for a margin of error. Furthermore, the maximum temperature was fixed as +220 °C considering the margin of error based on the simulation performed at an ambient temperature of +100 °C when surrounded by air. In addition, the voltage $V_{1}$ that included the margin ratio, ranged from 6.42 V (minimum) to 7.96 V (maximum). The voltages of $V_{7}$ and $V_{8}$ were chosen so that the circuit could determine whether the voltage of $V_{1}$ was in the normal range. As shown in Table 4, the output of the submersion detector was disabled when the RTDs exhibited a fault condition (open, short, defective circuit, etc.) and the self-diagnosis circuit output 0 V to inform the system of the fault.

### 2.4. Test Equipment Design

**Figure 9:**
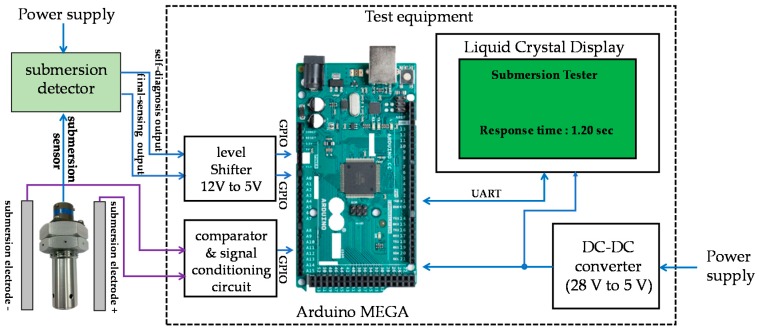
Test equipment of the submersion sensor.

As shown in Figure 9, the Arduino MEGA, which is a well-known open source hardware platform, was used to test the response time of the submersion detection. The test equipment used two separate submersion electrodes to get the exact time of submersion. It also had a signal conditioning circuit and a comparator in order to detect the submersion signal. The response time was displayed at a resolution of 1 ms on an LCD after the submersion signal was detected from the submersion electrodes. A UART (Universal Asynchronous Receiver Transmitter) was used as an interface with the LCD. Because the self-diagnosis output and final-sensing output was 12 V, a level shifter was used to step the 12 V signals down to 5 V signals for the Arduino MEGA.

## 3. Results

**Figure 10:**
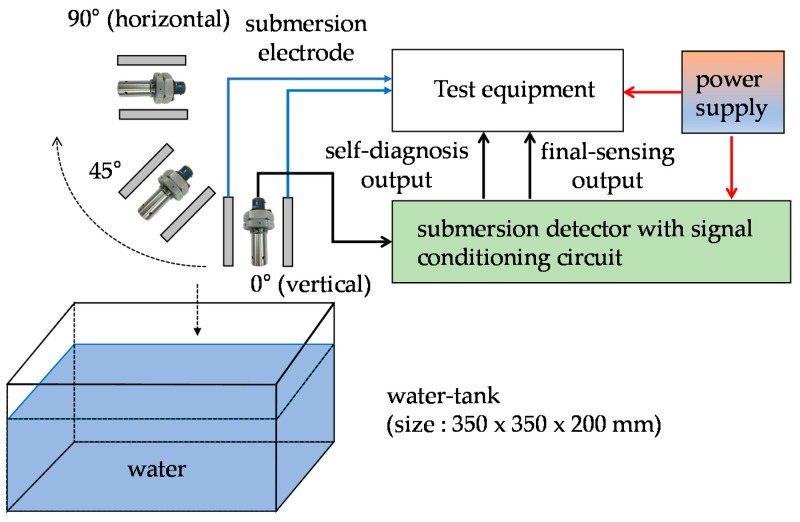
Test setup of the submersion sensor.

The four samples of the submersion detector configured by the proposed submersion detection sensor and signal conditioning circuit were fabricated. The static characteristics of the samples were tested to verify malfunctions in high humidity and rainfall conditions as shown in Figure 10. The submersion response time under dynamically varying conditions were tested in the directions of 0° (vertical), 45°, and 90° (horizontal) using fresh-water and salt-water.

### 3.1. Static Test

#### 3.1.1. Humidity Test

**Figure 11:**
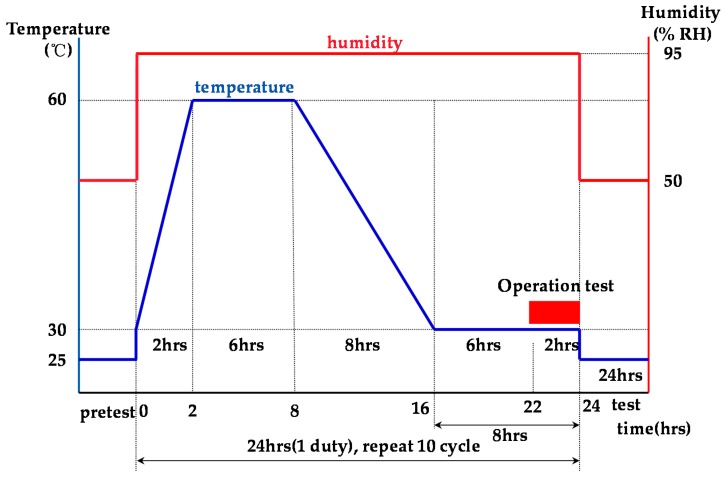
Test profile of humidity.

MIL-STD-810G is the U.S. military standard for environmental testing applied to military equipment and commercial products. Humidity tests were performed in accordance with Procedure II of the MIL-STD-810G Method 507.5 [26]. Furthermore, PSL-4J temperature and humidity chamber (ESPEC Corporation) was used as a humidity test system [27]. Under our test conditions, the temperature was 25−60 °C and the humidity was 95 %. The results of the test conducted with the profile shown in Figure 11 for a total of 240 h showed no malfunctions, and no submersion was detected as shown in Table 5.

#### 3.1.2. Rain Test

Rainfall tests were conducted in accordance with Procedure I and III of the MIL-STD-810G Method 506.4, and the results of the test showed no malfunctions in normal rain, blowing rain, and drip conditions as shown in Table 6.

### 3.2. Dynamic Characteristics: Submersion Sensing Response Time 

The submersion sensing response time of the dynamic characteristic was tested in accordance with MIL-STD-801G in an ambient temperature range of −40 to +71 °C. The temperature of fresh-water and salt-water was examined as shown in Table 7 and tested in the range of −1.7–32 °C. The test results are presented in Table 8 and Table 9 [28,29]. There was no change in the submersion sensing response time due to environmental temperature and submersion temperature, and the submersion sensing response time was measured in the range of 0.4–0.6 s. The reason the submersion sensing response time in the horizontal direction was about 0.1 s shorter than that for the vertical direction was thought to be that the inflow speed of water into the submersion detector was faster due to its structure. The response time obtained from the simulation with MATLAB was about 0.3 s. The difference of 0.1–0.3 s between the simulation and the experiment was thought to stem from the fact that the inflow speed of water to the submersion sensor and the time taken to fill the inside with water were not considered in the simulation.

### 3.3. Self-Diagnosis Test

The sensors attached to expensive systems may cause catastrophic effects on the system if a fault or malfunction occurs. Therefore, it is essential to have a self-diagnosis function to determine whether the sensor is in the normal condition. The test results of the proposed self-diagnosis function of the submersion detector is shown in Table 10. When the RTD sensor failed either because the circuit was open, or because of a short circuit, the error was detected under all conditions.

### 3.4. Repeatability Test

**Figure 12:**
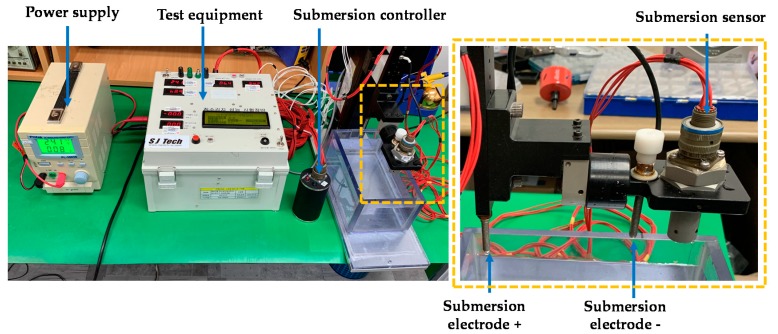
Experimental setup for the repeatability test.

Figure 12 shows the experimental setup that was prepared for the repeatability test. The test equipment shown in Figure 12 displays the response time, pass/fail result of submersion detection, and output voltages of the signal conditioning circuit for each test. The repeatability was tested through 1000 experiments with fresh water and salt water. There was no malfunction observed as shown in Table 11.

## 4. Discussion

We verified that the proposed submersion detection sensor operated on the thermal equilibrium principle, and thus had a very strong dependence on environmental factors, i.e., environmental temperature, submersion temperature, humidity, and rainfall conditions. Furthermore, it turned out that the submersion sensing response time was about 0.4–0.6 s, which was a little longer than those of existing submersion detection sensors. The malfunctions caused by external environmental conditions in practical applications are unacceptable, and the submersion sensing response time of 0.4–0.6 s is not an issue in most practical applications. Table 12 shows the comparison between the proposed submersion detection sensor and the existing submersion detection sensors.

## 5. Conclusions

In this study, two RTDs were used to propose a new submersion detection sensor that operates based on the thermal equilibrium principle. A signal conditioning circuit and a self-diagnosis circuit were also proposed. The transient heat transfer mechanism of the RTDs used in this submersion detection sensor was analyzed with MATLAB. Although the response time is slower than other submersion detection sensors, it is particularly robust against malfunctions caused by external environmental factors. It was verified that there were no malfunctions under the humidity and rainfall test conditions of MIL-STD-801G. Therefore, it can be useful for detecting the submersion of ships and aircraft exposed to the external environment, detecting submersion in low areas, and detecting submersion of major facilities. Because the RTD sensor used for the proposed submersion sensor was produced at a cost of tens of U.S. dollars, economic feasibility can be improved by using thin film type RTDs in the sensor. As future research work, we will conduct studies with the goal of reducing the size of the sensor and improving the submersion sensing response time.

## Figures and Tables

**Table 1:** Specification of the resistance temperature detectors (1PT100GO1020).

Operating Temperature Range [°C,F]	Shape: Cylinder Size: [mm]	Self-Heating Error [°C/mW] @ Air Flow V = 1 [m/s]	Response Time [Second]			
			Flowing Water V = 0.4 [m/s]		Flowing Air V = 1 [m/s]	
			Response 50%	Response 90%	Response 50%	Response 90%
**−220–400,** **(−365–750)**	**Radius: 1** **Length: 10**	0.26	0.14	0.35	7.0	21.0

**Table 2:** Values of the Biot number in air and submersion conditions.

Condition	Air = 10 [W/m^2^ °C]	Air = 30 [W/m^2^ °C]	Air = 50 [W/m^2^ °C]	Water = 500 [W/m^2^ °C]	Water = 700 [W/m^2^ °C]
k [W/m^2^ °C]	8.5	8.5	8.5	15	15
$B_{i}$	0.0005347	0.0016041	0.0026735	0.01515	0.02121

**Table 3:** Simulation and measurement results of the temperature and voltage of resistance temperature detectors (RTDs) with the signal conditioning circuit in Figure 6.

Condition	Simulation Results			Measurement Results		
	Before Submersion	After Submersion	Difference	Before Submersion	After Submersion	Difference
temperature of $RTD_{H}$ [°C]	55	39	16	55.6	39.3	16.3
resistance of $RTD_{H}$ [Ω]	121.17	115.01	6.16	121.40	115.13	6.27
voltage of $RTD_{H}$ [V] @$\;V_{1}$	7.02	6.90	0.12	7.04	6.92	0.11
temperature of $RTD_{A}$ [°C]	20.0	20.0	0	20.6	20.3	0.3
resistance of $RTD_{A}$ [Ω]	107.7	107.7	0	107.9	107.8	0.1
voltage of $RTD_{A}$ [V] @$\;V_{2}$	3.25	3.25	0	3.25	3.25	0
output of $U_{4D}$ [V] @ $V_{3}$	5.75	5.75	0	5.77	5.77	0
output of $U_{5A}$ [V] @ $V_{4}$	1.26	1.14	0.12	1.26	1.15	0.11
output of $U_{5B}$ [V] @ $V_{5}$	7.20	6.54	1.12	7.23	6.56	0.67
reference of $U_{5B}$ [V]	6.8	6.8	0	6.78	6.78	0
sensing margin [V]	0.40	0.26	-	0.45	0.21	-
output of $U_{5c}$ [V] @ $V_{6}$	0 V (“low”)	12 V (“high”)	-	0 V (“low”)	12 V (“high”)	-
Sensing output [V]	0 V (“low”)	12 V (“high”)	-	0 V (“low”)	12 V (“high”)	-

**Table 4:** Logic table of the self-diagnosis circuit.

Condition		Temp. of *RTD_H_* [°C]	Resistanceof *RTD_H_* [Ω]	*V*_1_ [V]	*V*_7_ [V]	*V*_8_ [V]	Self-Diagnosis Output	Sensing Output	Final Sensing Output
**Normal**	low temp	−20	92.3	6.42	8.35	6.13	12 V	0 V	0 V
	high temp	+220	184.7	6.42	8.35	6.13	12 V	12 V	12 V
malfunction	RTD open	-	-	12	8.35	6.13	0 V	12 V	0 V
	RTD short	-	-	0	8.35	6.13	0 V	12 V	0 V

**Table 5:** Humidity test result.

Test Condition	Sample 1	Sample 2	Sample 3	Sample 4
MIL-STD-801G Method 507.5 Procedure Ⅱ	submersion not detected	submersion not detected	submersion not detected	submersion not detected

**Table 6:** Rainfall test results.

Test Condition	Description	Sample 1	Sample 2	Sample 3	Sample 4
MIL-STD-801G Method 506.4 Procedure Ⅰ	rain and blowing rain	submersion not detected	submersion not detected	submersion not detected	submersion not detected
MIL-STD-801G Method 506.4 Procedure Ⅱ	Drip	submersion not detected	submersion not detected	submersion not detected	submersion not detected

**Table 7:** Water Temperature.

Water	Minimum Temperature [°C]	Maximum Temperature [°C]	psu (Practical Salinity Unit)
Fresh water	1	29	-
Salt water	−1.7	31.7	maximum 40 psu [30]

**Table 8:** Submersion sensing response time of fresh-water.

Air Temperature [°C]	Fresh-Water Temperature [°C]	Submersion Direction	Sample 1 [Second]	Sample 2 [Second]	Sample 3 [Second]	Sample 4 [Second]
−40	2	0° (vertical)	0.52	0.56	0.51	0.50
		45°	0.34	0.50	0.52	0.55
		90° (horizontal)	0.40	0.38	0.49	0.42
	32	0° (vertical)	0.47	0.46	0.53	0.43
		45°	0.48	0.39	0.32	0.45
		90° (horizontal)	0.39	0.42	0.43	0.39
0	2	0° (vertical)	0.41	0.59	0.54	0.40
		45°	0.33	0.45	0.47	0.44
		90° (horizontal)	0.35	0.42	0.40	0.39
	32	0° (vertical)	0.56	0.46	0.51	0.53
		45°	0.45	0.43	0.47	0.39
		90° (horizontal)	0.37	0.39	0.44	0.37
71	2	0° (vertical)	0.49	0.55	0.50	0.58
		45°	0.42	0.44	0.49	0.45
		90° (horizontal)	0.35	0.42	0.38	0.41
	32	0° (vertical)	0.51	0.59	0.58	0.53
		45°	0.49	0.45	0.48	0.49
		90° (horizontal)	0.36	0.44	0.43	0.38

**Table 9:** Submersion sensing response time of salt-water (40 psu).

Air Temperature [°C]	Salt-Water Temperature [°C]	Submersion Direction	Sample 1 [Second]	Sample 2 [Second]	Sample 3 [Second]	Sample 4 [Second]
−40	2	0° (vertical)	0.55	0.51	0.44	0.57
		45°	0.43	0.49	0.38	0.44
		90° (horizontal)	0.32	0.36	0.35	0.35
	32	0° (vertical)	0.52	0.48	0.56	0.55
		45°	0.44	0.47	0.39	0.45
		90° (horizontal)	0.39	0.41	0.38	0.40
0	2	0° (vertical)	0.50	0.47	0.58	0.41
		45°	0.43	0.55	0.46	0.44
		90° (horizontal)	0.39	0.40	0.30	0.35
	32	0° (vertical)	0.54	0.49	0.46	0.54
		45°	0.48	0.41	0.39	0.48
		90° (horizontal)	0.45	0.33	0.38	0.32
71	2	0° (vertical)	0.56	0.47	0.49	0.56
		45°	0.51	0.42	0.53	0.37
		90° (horizontal)	0.48	0.35	0.36	0.35
	32	0° (vertical)	0.38	0.52	0.51	0.54
		45°	0.40	0.43	0.46	0.43
		90° (horizontal)	0.35	0.38	0.37	0.35

**Table 10:** Self-diagnosis test results.

Test Condition	Self-Diagnosis Output (Normal: 12 [V], Detected: 0 [V])			
	Sample1	Sample2	Sample3	Sample4
Normal	12 V	12 V	12 V	12 V
$RTD_{H}$ open	0 V	0 V	0 V	0 V
$RTD_{H}$ short	0 V	0 V	0 V	0 V
$RTD_{A}$ open	0 V	0 V	0 V	0 V
$RTD_{A}$ short	0 V	0 V	0 V	0 V

**Table 11:** Repeatability test result (atmosphere 20 °C).

Water	Temperature [°C]	Submersion Direction	Repeatability Test Condition	Test Result
Fresh-water	2	0°, 90°	1000 times	Pass
	32	0°, 90°	1000 times	Pass
Salt-water with 40 psu	2	0°, 90°	1000 times	Pass
	32	0°, 90°	1000 times	Pass

**Table 12:** Comparison with existing submersion sensors.

	Malfunction Possibility	Self-Diagnosis	Response Time [sec]	Electro Magnetic Interference (EMI) Influence
Proposed submersion sensor	none	possible	0.4–0.6	none
Resistive electrode submersion sensor	medium	impossible	less than 0.1	none
Capacitive submersion sensor	high	possible	less than 0.1	yes
Optical submersion sensor	high	possible	less than 0.1	none
